# The role of cognition in cost-effectiveness analyses of behavioral interventions

**DOI:** 10.1186/1478-7547-10-3

**Published:** 2012-03-01

**Authors:** Rilana Prenger, Louise M Braakman-Jansen, Marcel E Pieterse, Job van der Palen, Erwin R Seydel

**Affiliations:** 1 University of Twente, Department of Psychology, Health and Technology, PO Box 217, 7500 AE Enschede, The Netherlands; 2 Medisch Spectrum Twente Hospital, Department of Clinical Epidemiology and Statistics, PO Box 50 000, 7500 KA Enschede, The Netherlands

**Keywords:** Cost-effectiveness, Behavior, Decision modeling, Psychological theory, Cognitive parameters, Health promotion

## Abstract

**Background:**

Behavioral interventions typically focus on objective behavioral endpoints like weight loss and smoking cessation. In reality, though, achieving full behavior change is a complex process in which several steps towards success are taken. Any progress in this process may also be considered as a beneficial outcome of the intervention, assuming that this increases the likelihood to achieve successful behavior change eventually. Until recently, there has been little consideration about whether partial behavior change at follow-up should be incorporated in cost-effectiveness analyses (CEAs). The aim of this explorative review is to identify CEAs of behavioral interventions in which cognitive outcome measures of behavior change are analyzed.

**Methods:**

Data sources were searched for publications before May 2011.

**Results:**

Twelve studies were found eligible for inclusion. Two different approaches were found: three studies calculated separate incremental cost-effectiveness ratios for cognitive outcome measures, and one study modeled partial behavior change into the final outcome. Both approaches rely on the assumption, be it implicitly or explicitly, that changes in cognitive outcome measures are predictive of future behavior change and may affect CEA outcomes.

**Conclusion:**

Potential value of cognitive states in CEA, as a way to account for partial behavior change, is to some extent recognized but not (yet) integrated in the field. In conclusion, CEAs should consider, and where appropriate incorporate measures of partial behavior change when reporting effectiveness and hence cost-effectiveness.

## Background

Resources in health care are generally limited. Consequently, funding priorities have to be set, preferably based on information that concerns the effectiveness and efficiency of available alternatives. In the health care systems in developed countries, cost-effectiveness analyses (CEAs) have become an accepted method to assess and improve the efficiency of pharmaceutical treatments [1,2] as in the field of health psychology and health promotion.

Performing a CEA on a health promotion intervention, however, has some implications for the CEA methodology, compared to pharmaceutical interventions. Generally, health promotion interventions are designed to accomplish behavior change. CEAs of these interventions typically focus on objective behavioral data, i.e. physical endpoints like weight loss or biochemically validated smoking cessation [3,4]. In reality, though, behavior change is a complex process in which several steps towards success are taken, including changes in cognitive antecedents of behavior. Any progress in behavior change without accomplishing full behavior change may also be considered as a beneficial outcome of an intervention, assuming that this increases the likelihood to achieve successful behavior change eventually [5]. Adding partial effects can therefore improve the structure of CEA models in the field of health promotion. Butler et al. concluded from their study on smoking cessation that *'...focusing on quitting alone may understate efficiency on a wider range of related objectives such as reducing addiction or moving smokers towards the 'action' end of the stages of change continuum' *[6]. Similarly, Wagner & Goldstein argued that analysts who conduct a CEA of a behavioral intervention should not focus solely on people who successfully changed their behavior, but should also consider partial behavior change. Any progress in the process of behavior change caused by the intervention can then be included as a partial behavior change that may predict full behavior change in the future. Conversely, failing to include such partial effects in CEAs may bias the results [3].

Thus, in order to predict full behavior change after the study period ends, 'intermediate' outcomes of behavior change could be measured. Subsequently, modeling techniques like decision trees and Markov models are required to model these intermediate outcomes to final outcomes. Including intermediate outcomes in CEAs, though, has been subject of a large literature. The main counter argument is that a treatment can improve intermediate endpoints without (yet) improving the final health outcome [7]. Also, in these intermediate endpoints, important aspects of the intervention may not be caught. Thus, reliance on solely intermediate outcomes may over- or underestimate final outcomes [1]. Ultimately, the validity of intermediate outcomes in CEAs depends on the strength of the evidence that links the intermediate to the final health outcome of interest. The underlying assumption of intermediate or surrogate outcomes is that an intervention's effect on these end points predicts an effect on the outcome of interest. Although the terms 'surrogate' and 'intermediate' are sometimes used synonymously, there is a clear distinction. A surrogate outcome is not necessarily an intermediate step in a *causal *pathway, this in contrast to an intermediate outcome, and avoids any implication of causality [7]. Examples are prostate-specific antigen in prostate cancer as the indication of an advanced tumor stage [8] and morbidity as surrogate for mortality. In this case a causal relationship between intermediate, partial behavior change and full behavior change is a precondition to be able to predict future behavior. This precludes the use of surrogate outcomes within the scope of this paper.

Cognitive determinants of behavior can predict health behavior change and progression (or decline) in these determinants can also been seen as partial behavior change. These outcome measures are derived from theories, which are used to explain and predict behavior (change) and to guide the development and refinement of health promotion and education efforts [9]. Cognitive outcome measures are antecedents of behavior change, and can therefore be measured at some intermediate time point to predict health behavior in the future. Examples are psychological constructs such as attitudes, self-efficacy, risk perception, and social support. Previous research has demonstrated convincingly that several theories are successful in predicting a wide range of health behaviors [10,11].

The empirical basis for these constructs can be found in for example the Transtheoretical model of behavior change. This stage-oriented model describes the readiness to change [12]. It has been widely adopted for numerous health behaviors, but was originally designed to describe addictive behaviors and was based on research of self-initiated quit attempts by smokers [13]. A number of qualitatively different, discrete stages are key constructs of the Transtheoretical model. It provides an algorithm that distinguishes six stages, of which five are often used: 1) pre-contemplation (e.g. no intention to quit smoking within the next six months); 2) contemplation (e.g. intending to quit smoking within the next six months, but not within the next month); 3) preparation (e.g. intending to quit smoking within the next 30 days [13]); 4) action (e.g. being abstinent for less than six months); and 5) maintenance (e.g. being continuously abstinent from smoking for more than six months). The first three *pre-action *stages reflect stages of partial behavior change. Each pre-action stage provides probabilities for the *actual *transition to the fourth stage, the 'action stage' in which full behavioral change is achieved. The stage algorithm has been developed on the basis of empirical findings. Usually, attempts to modify (addictive) behavior are not immediately successful. With smoking, for example, successful quitters make an average of three to four attempts and go through a spiral pattern of several cycles before they reach long term abstinence. Relapse and recycling through the stages therefore occur quite frequently as individuals attempt to modify or cease addictive behaviors [13]. To classify participants according to their stage-of-change, questionnaires have been developed to assess readiness to change in individuals. Another example is the Theory of Planned Behavior [14], which is one of the most influential theories and has been used to predict many health behaviors successfully. It proposes that certain behavior can be predicted by a person's intention to perform that behavior. This behavioral intention in fact is closely related to the 'stages-of-change'-construct. According to the theory, the behavioral intention in turn is determined by a positive attitude towards smoking cessation, a high perceived behavioral control to refrain from smoking, and a high perceived social norm to stop smoking [15]. These psychological constructs are generally assessed with multiple-item questionnaires using Likert type scales. Self-reported scores of respondents are summated to a score on a unidimensional scale. An important distinction between stage theories such as the Transtheoretical model and social cognitive theories such as the Theory of Planned Behavior is that the former classifies subjects according to a discrete (dichotomous) stages-of-change algorithm, while the latter consists of dimensional variables that predict and explain behavior change.

Overall, the aforementioned social-cognitive determinants could be used as outcome measures reflecting partial behavior change which could be incorporated in CEAs - assuming adequate predictive value for the study of interest. This requires the combined expertise from the fields of health psychology and health economics. Although these disciplines share many goals (e.g., increasing healthy behaviors [16]), collaboration has been limited on this particular issue.

The aim of this explorative review is to identify CEAs of behavioral interventions in which cognitive outcome measures of behavior change are analyzed. The goals of the present review are: 1) to identify which cognitive outcome measures of behavior change can be distinguished in CEAs; and 2) to evaluate whether and how these outcomes are incorporated in CEAs.

## Methods

All studies that conducted a cost-effectiveness (CEA), cost-utility (CUA) or cost-benefit analysis (CBA) and additionally included or reported cognitive outcome measures of behavior change were considered for inclusion in this review. Interventions to accomplish behavioral change were compared to usual care or to an alternative intervention in these selected analyses.

Electronic databases (ScienceDirect, Scopus, Medline, Web of Science, HEED, EMBASE and PsycInfo) were searched for English or Dutch language publications that were published before May 2011 by standardized search strategies. The core search strategy used for this review was as follows: 1) ICER or cost-effectiveness or cost-utility or cost-benefit; 2) 1 and health; 3) 2 and behav*; 4) 3 and (model* or cogn*). Due to the exploratory character of this review, a broad search strategy was employed. Titles and abstracts of all citations generated from the search were assessed meeting inclusion and exclusion criteria to identify eligible publications. To identify additional publications, hand searches of reference lists were conducted. Studies that report costs and effects in a disaggregated way were excluded as this review aims to explore the methodology of applying cognitive outcome measures in CEA.

Data from eligible studies were entered into a matrix. Collected characteristics were the author(s) and year of publication, the study topic, a short description of the intervention, the effectiveness measure for CEA, the cognitive (intermediate) outcome measures of behavior change, the type of behavioral model used and a short description of the application of the cognitive outcome measure in the study (Table 1). The elements of the economic evaluations were not assessed in this review, as the focus was not on the actual final results of the analyses. Additionally, sufficient evidence for the validity of included cognitive intermediate outcomes of behavioral change needs to be available. Therefore, the validity was examined by considering the theoretical foundation of the reported cognitive outcome measures. If these are derived from empirically well-tested theories, a causal relation may be assumed. For this review, we consider this to be a prerequisite for a cognitive intermediate outcome to be valid.

**Table 1:** Characteristics of included studies

Authors	Topic	Intervention	Effectiveness measure	Cognitive outcome measures	Behavioral model used	Application of cognitive outcome measures
Butler et al. 1999 [6]	Smoking cessation	Motivational consulting with brief advice	Smoking cessation, reduction in addiction and quit attempts	Stages-of-change	Transtheoretical model, self-efficacy theory	Effectiveness was calculated per stage-of-change at baseline and cognitive outcomes were used as secondary outcome measures

Crane et al. 2000 [17]	Mammography screening	Multiple outcall approach	Mammography screening	Stages-of-change, attitudes and knowledge	Transtheoretical model	Cognitive outcome measures were used to describe the theoretical foundations of the intervention and as secondary outcome measures

Emmons et al. 2005 [18]	Smoking cessation	Peer counseling or self-help intervention	Smoking cessation	Stages-of-change, self-efficacy, perceived vulnerability, social support and knowledge	Transtheoretical model, social ecological model	Cognitive outcomes were used as secondary outcome measures

Kyle et al. 2008 [19]	Sun protection	Sun protection education for young children	Nonfatal cases and premature mortalities averted and QALYs saved	Knowledge, attitude & intention	No theoretical foundation in model	Cognitive outcomes were used as secondary outcome measures

Lo et al. 2009 [20]	Self-care behavior for stoma patients	Multimedia learning education program	Knowledge, attitude and behavior of self-care	Knowledge and attitude of self-care	No theoretical foundation in model	The effectiveness measure was a combined score of knowledge, attitudes and behavior of self-care

Oldenburg et al. 1995 [21]	CVD risk reduction	CVD risk reduction programs	Unweighted CVD lifestyle risk scores	Stages-of-change	Transtheoretical model, social learning theory	Stages-of-change were used to appoint follow-up periods

Pyne et al. 2005 [22]	Patient receptivity to anti-depressants	Evidence-based primary-care depression intervention	QALYs	Attitude	No theoretical foundation in model	Two separate CE ratios were calculated for both negative and positive attitudes toward antidepressants

Rasu et al. 2010 [23]	Weight management	Internet-based weight management program	Change in body weight, a weight change of 5% or more, and waist circumference.	Social pressure	No theoretical foundation in model	A CE ratio was calculated for each additional point gain on the Social Pressure subscale, indicating increased confidence in managing social pressures to eat

Saywell et al. 1999 [24]	Compliance with mammography screening	Counseling strategies	Increase in mammography rate	Intention to screen	Health Belief Model	Cognitive outcome was used as secondary outcome measure

Sims et al. 2004 [25]	Changing GP's behavior	Organized approach to exercise counseling	Amount of patients screened, activity, accruing health benefit, DALYs and premature deaths averted	Knowledge & attitudes	No theoretical foundation in model	Cognitive outcomes were used as secondary outcome measures

Smith et al. 2007 [26]	Smoking cessation	Multi component expert system intervention	Quit smoking	Stages-of-change	Transtheoretical model	An ICER was calculated that incorporated partial behavioral change as measured by the stages-of-change

Sood & Nambiar 2006 [27]	HIV/AIDS prevention	Entertainment-education-based mass media campaign	Condom use frequency and changes in cognitive parameters of behavior change	Knowledge, gender attitudes, & perceived risk	Multiple stage models of behavior change	Cost-effectiveness was calculated for condom use frequency and additionally for changes in the three cognitive outcome measures

Note. *Year *year of publication, *GP *general practitioner, *CEA *cost-effectiveness analysis, *CE ratio *cost-effectiveness ratio, *ICER *incremental cost-effectiveness ratio, *CVD *cardiovascular disease, *QALY *quality adjusted life year, *DALY *disability adjusted life year

## Results

Of the 5,916 studies identified, 137 were qualified for the final selection. After the inclusion and exclusion criteria were applied by the reviewers, 12 CEAs and CUAs were identified that reported cognitive outcome measures of behavior change and therefore were eligible for review. Seventy eight studies were excluded for not reporting data on cognitive outcome measures of behavior change. Three studies were excluded as the function of the cognitive outcome measures was solely for design purposes of the intervention and not the CEA. In six studies the interventions were not aimed at behavioral change and in six other studies the authors had retrieved their results through meta-analyses. Furthermore, eight publications consisted of a study protocol or model development and in three studies there were no interventions described. Also, 21 studies were excluded for only reporting effects, and for reporting cost and effects separately.

In Table 1 details of the 12 included studies are shown. The included studies can be assigned to two categories describing the application of the cognitive outcome measures in these studies. The first category describes studies that integrated cognitive outcome measures in CEA. The second category contains studies that reported cognitive outcomes which were merely used as secondary outcomes of the intervention. In this last category of studies the cognitive outcome measures were not related to CEA.

### Incorporated in CEA

Four studies integrated cognitive outcome measures of behavior change in the CEA [22,23,26,27]. First, one study modeled partial behavior change measured by stages-of-change construct (Transtheoretical model) into the ICER. Smith et al. studied the incremental (cost-) effectiveness of a computerized smoking cessation intervention for primary care physicians. The mean ICER was $1,174 per LYS ($869 per QALY). However, the authors additionally considered the intervention impact on progression in stages-of-change. By advancing a smoker's stage-of-change and adjusting for a 45% relapse rate, partial behavior change was incorporated in the ICER [26]. Consequently, this ratio declined 15% to $999 per LYS ($739 per QALY).

Second, three studies were found that calculated different ICERs for effects on cognitive outcome measures of behavior change. These papers applied a fundamentally different approach than Smith et al..: in these studies between-group differences in ICER outcomes were calculated by performing CEAs within subgroups [22] or separate ICERs were calculated for cognitive outcome measures in addition to the ICER for the behavioral outcome measure [23,27].

Pyne et al. studied the impact of patient treatment attitudes on the cost-effectiveness of healthcare interventions. The cognitive outcome measure attitude has been described as part of many social cognitive theories (e.g., Theory of Planned Behavior). The study estimated the impact of patient receptivity to antidepressant medication on the cost-effectiveness of an evidence-based primary-care depression intervention. Among patients receptive to antidepressants, the mean incremental cost-effectiveness ratio (ICER) was $5,864 per QALY, and was negative for patients non receptive to antidepressants [22]. Rasu et al. evaluated the cost-effectiveness of a behavioral internet treatment program for weight management compared with usual care in a diverse sample of overweight adults in the United States Air Force. The ICERs for the primary outcomes indicated that the costs to lose one additional kilogram of weight, lose one additional centimeter of waist circumference, and make one additional 5% or more weight change were $25.92, $28.96 and $3.12 respectively. Additionally, an ICER was calculated for the cognitive outcome measure social pressure. For each additional point gain on the Social Pressure subscale (Weight Efficacy Lifestyle questionnaire), where increasing scores indicated increased confidence in managing social pressures to eat, the cost was $37.88 [23]. Sood & Nambiar examined the impact of exposure to entertainment-education-based mass media campaigns to prevent HIV. The cost-effectiveness was calculated for different components of the campaign for the behavioral outcome condom use. Additionally, cost-effectiveness was calculated for changes on measures of the cognitive outcome measures knowledge, gender attitudes and perceived risk [27].

In contrast to the other studies reported above [22,23,27], yet another approach is used to account for partial behavior change by Oldenburg et al. [21]. They focus on the difference between the two 'action stages', by comparing short-term behavior change (< 6 months) as outcome with long-term (> 6 months) behavior change. Thus, these authors did not predict future behavior change by modeling cognitive outcome measures like Smith et al., but they collected outcomes at six and 12 months for the interventions and calculate ICERs at both stages. In other words, they examined the economic aspects of the action and maintenance stage of lifestyle change to reduce cardiovascular disease. Instead of using the *patient's *stage-of-change as Smith et al. did in their study, they calculated different ICERs of a *program's *stage-of-change. Results showed that depending on the follow-up period, cost-effectiveness results varied. For the analysis of cardiovascular risk reduction during the 'action phase' (six months), the least expensive program, health risk assessment (HRA), was not effective in initiating change at all, and the most expensive program in the base assessment of costs, behavioral counseling plus incentives (BCI), was the least cost-effective. Behavioral counseling (BC) cost only marginally less than BCI, but proved to be almost twice as clinically effective and was considerably more cost-effective. Risk factor education (RFE) cost half that of BCI, yet was equally effective in terms of lifestyle change and was at a similar level of cost-effectiveness to BC. However, when the maintenance of the effects of the interventions was assessed 12 months after the start of the interventions (maintenance stage), the cost-effectiveness of the programs differed from the costs at six months follow-up. Only BC demonstrated significant risk reduction with little loss of cost-effectiveness from the earlier results. Both BCI and RFE were ineffective in sustaining change. For the BC intervention there was minimal relapse up to the 12-months follow-up and consequently emerged as the most cost-effectiveness intervention on the longer term. This study reveals that behavioral interventions may turn out to be more cost-effective when the probability of maintenance of behavior change is increased (or relapse to pre-action stages-of-change is prevented) [21].

### Secondary outcome measures

In the second category cognitive outcome measures were reported as secondary outcomes of the intervention, without relating these outcome measures to the CEA. In seven studies the cognitive outcome measures of behavior change served as secondary outcome measures of the intervention [6,17-20,24,25]. The stages-of-change served as secondary outcomes in Butler et al. They assessed whether the effects of motivational consulting on smoking cessation were modified by subject's prior stage of change [6]. Also, in the study of Crane et al. the stages-of-change for mammographic screening served as a secondary outcome measure as well as for intervention design. In addition, knowledge, attitudes and perceived barriers toward mammographic screening were measured [17]. Emmons et al. report on the outcomes of a smoking cessation intervention for smokers in the Childhood Cancer Survivors Study. Their interest was the extent to which several psychosocial factors were predictive of smoking cessation outcomes. Self-efficacy, stages-of-change, perceived vulnerability, social support and knowledge were also measured besides the quit rates for smoking [18]. Kyle et al. report the results of an economic analysis on a school-based sun safety education program. Secondary outcomes were knowledge, attitudes and intention towards sun protection behaviors [19]. Lo et al. compared the costs and effectiveness of enterostomal education using a multimedia learning education program and a conventional education service program. The effectiveness measure consisted of a combined score of knowledge of self-care, attitude of self-care and behavior of self-care. The cost measures for each patient were: health care costs, costs of the multimedia learning education program, and family costs [20]. Cost-effectiveness of five combinations of physician recommendation and telephone or in-person individualized counseling strategies for increasing compliance with mammographic screening was examined by Saywell et al. Besides an increase in mammography rate, the intention to screen was measured [24]. Sims et al. conducted a CEA on the 'Active Script Program' that aimed to increase the number of general practitioners who deliver appropriate, consistent, and effective advice on physical activity to patients. General practitioners' knowledge and attitude towards providing such advice were the cognitive parameters used as secondary outcome measures [25].

### Cognitive parameters as theory-based intermediate outcomes

For all studies, the theoretical foundation of the cognitive outcome measures was judged, as reported in the selected articles. Five studies measured cognitive outcome measures of behavior change before and after the intervention, without explicitly describing a theoretical foundation of these outcome measures [19,20,22,23,25]. It is therefore not clear from these studies, whether the cognitive outcomes reflect true intermediate outcome measures. Kyle et al. measured knowledge, attitude and intention towards sun protection behavior among young children [19]. Lo et al. measured knowledge and attitudes of self-care behavior for stoma patients [20]. Pyne et al. reported attitude towards antidepressant medication as parameter of major depression [22]. Rasu et al. measured social pressure in weight management which indicates the confidence in managing social pressures to eat [23]. Sims et al. measured knowledge and attitude of general practitioners regarding counseling patients on physical exercise [25].

Five studies reported different stages of the Transtheoretical model as parameters of behavior change [6,17,18,21,26]. These studies reported stages-of-change towards smoking cessation, except Crane et al., who reported stages-of-change towards participation in mammographic screening.

Two studies reported other theories of behavior change that provided cognitive outcome measures for their studies [24,27]. Saywell et al. conducted a study on mammographic screening and additionally measured the intention to screen, which was derived from the Health Belief Model [24]. Sood & Nambiar measured the parameters HIV knowledge, gender attitudes and perceived risk of HIV/AIDS, which were constructs of multiple stage models of behavior change, i.e. McGuire's hierarchy of effects, the stages-of-change model, steps to behavior change, Rogers's innovations decision model and Kincaid's ideation theory [27].

## Discussion

Current CEA research of behavioral interventions predominantly relies on behavioral outcome measures. However, these do not take into account delayed behavior change that may occur after the follow-up period ends, and may consequently underestimate cost-effectiveness of psychological interventions. Furthermore, RCTs in the field of health promotion often are limited by a relatively short follow-up, increasing the likelihood of missing delayed effects. To remedy this, delayed intervention effects should somehow be incorporated in CEA. A number of empirically well-tested social-cognitive theories are available that enable prediction of future behavior change based on valid cognitive outcome measures, such as self-efficacy expectations [14,28-30]. Progression on these cognitive outcome measures can be seen as a beneficial outcome of an intervention, assuming that such a cognitive progression precedes behavior change. By broadly examining literature we explored whether there is potential for including cognitive outcomes in CEAs of health promotion, and what techniques are known to perform this. We found that the use of cognitive outcome measures in calculating ICERs is to some extent recognized, but is still in its infancy. The cognitive outcomes in the studies found served mainly as secondary outcome measures of the intervention and were not considered for CEA, except for four studies [22,23,26,27]. Two different frameworks for incorporating cognitive outcome measures preceding behavior change were distinguished from these results.

In the first framework the projected final outcomes are modeled based on cognitive outcome measures of behavior change. In the study of Smith et al. cognitive outcome measures were used to make a prediction of future behavioral change over time as a consequence of the intervention [26]. Besides modeling the stage-of-change, Smith et al. also adjusted for future relapse of quitters to smoking in the CE ratio. In spite of this conservative approach, results showed a 15% decline of the CE ratio compared to the ratio that included only observed quitters at the end of the study period.

In the second framework cognitive outcome measures of behavior change are simply applied as alternative or secondary intervention outcomes in a CEA. Three studies qualified for this category [22,23,27]. These did not include partial behavior change by predicting future behavior change in the CEA as shown by Smith et al., but calculated ICERs for cognitive parameters as outcome measure of the intervention. Thus, these studies calculated different ICERs for different cognitive states. Importantly, both frameworks assume that improvements in cognitions eventually result in behavioral change. When an intervention results in significant changes in valid cognitive intermediate outcomes, it is assumed to be more likely that behavior change will occur later on as a result of this cognitive change. However, in contrast to the first framework, this assumption remains more implicit in this second framework. It would be informative to include separate ICERs for significant cognitive outcome measures in addition to or even as a substitute for the original ICER. Moreover, as the study by Smith et al. showed, incorporating cognitive outcome measures in CEA may produce results deviating from standard CEA methods that do not directly recognize the effect of behavioral change. This emphasizes the need to further explore the role of cognitive outcome measures in CEA. Also, it is unclear which framework (modeling future behavior change or calculating ICERs for cognitive outcomes) is preferred under which conditions. The approach of Smith et al. seems potentially more promising as it is a more sophisticated method to incorporate partial behavioral change. It also seems a more transparent approach as it makes the assumption that changes in cognitions eventually result in behavioral change more explicit, and enables sensitivity analyses of the parameters in the model. Moreover, this approach takes one step further: in this case cognitive outcome measures are used to estimate future behavior change in a prognostic model. This makes it also more demanding and complex, as the predictive value of the cognitive outcome measures is crucial for the validity of the results. A strong theoretical model can help to justify the choice for cognitive outcome measures as intermediate outcomes. Concerning the psychological theories described in this review, there has been some discussion in literature about the predictive validity of the Transtheoretical model. However, this discussion mainly concerns its supposed usefulness for designing stage-based, tailored interventions with superior effectiveness [31-33]. It has been the predictive validity of the stages-of-change construct itself that has received high empirical support [34,35]. Also, literature on, for example, the Theory of Planned Behavior provides ample evidence supporting the use of this theory for predicting behavior [10]. Other empirically well-supported health behavior theories are the Health Belief Model [28], the Theory of Reasoned Action [29], and Social Cognitive Theory [30]. There are models specific to behavioral areas such as safer sex [36] and alcohol use [37], as are integrated theories combining constructs from multiple theories [38,39]. Overall, many theories are available in literature that describe and predict health behavior change [40]. However, as the predictive value of cognitive constructs from these models will not be perfect, like most prediction models in CEA literature, sensitivity analyses remain essential in such economic evaluations in order to assess reliability of CEAs.

There are also some methodological issues that need to be considered. Firstly, the Transtheoretical model [13] distinguishes different stages of behavioral change and empirical data provide transition probabilities to predict movement through these stages. By means of Markov modeling, transition probabilities of moving to and from the 'action' or 'maintenance' stage, can predict the percentage of additional quitters and additional relapsers on the long term. For incorporating partial behavior change like Smith et al. [26], these additional quitters and relapsers can be added to or subtracted from those who already have accomplished full behavioral change at follow-up. For smoking cessation, the effects of the intervention will probably increase (as long as the rate of future quitters exceeds the rate of future relapsers), while the costs of the intervention remain constant. Consequently, the CE ratio decreases. However, for other behaviors than smoking, the empirical support for transition rates may not be equally robust. Furthermore, the transition probabilities may depend on several context variables, such as the population, the comparative intervention and the exact point in time at which behavioral change (or relapse) may occur. Lastly, the stage-oriented Transtheoretical model classifies individuals into discrete states. This enables the use of a Markov model, as this technique is based on multiple health states [41]. Many research is available describing the transition probabilities for the stages of change of the Transtheoretical model. The Theory of Planned Behavior does not distinguish qualitatively different states, but provides a multidimensional change continuum. In Markov modeling, this would require an almost indefinite number of health states. Probably, other decision analytic techniques like discrete event simulation may be needed to model continuous cognitive outcome measures to future behavioral change [42].

This raises another methodological issue. Scales used for cognitive outcome measures are usually based on 5-point Likert scales, which are in principle considered to produce ordinal data. However, there appears to be consensus in methodological literature that analyses based on 5-point scales in general result in findings similar to data obtained with interval scales, and may therefore be accepted in such analytical techniques [43-45].

Accounting for cognitive outcome measures of behavior may be advisable for several reasons. Firstly, as already outlined in the introduction, it is likely that delayed effects of behavioral interventions may occur due to relatively short follow-up periods. Ignoring delayed effects, may negatively bias CEA outcomes and, as a result, cost-effectiveness of behavioral interventions could be underestimated with current methodology. Oldenburg et al. already showed that cost-effectiveness results shift when exploring different follow-up periods, due to delayed effects and relapse [21]. Also, a review of Richardson et al. on cost-effectiveness of interventions to support self-care concluded that drawing general, reliable conclusions about the cost-effectiveness is problematic due to short follow-up periods [46]. Secondly, effectiveness data from existing trials that were not originally developed with the aim of a CEA are often unsuitable for CEAs due to a lack of adequate behavioral endpoints. However, if cognitive outcomes can serve as intermediate outcomes of behavioral change, these may be used in addition to or even as a substitute for current effectiveness outcomes. Obviously, which specific cognitive parameters are valid intermediate outcomes, depends on the behavior of interest and should be explored before consideration in CEA. Potentially, including cognitive outcome measures can make many more health promotion programs available for health economists to evaluate cost-effectiveness.

Considering limitations, some are of note. Firstly, the focus of this review is restricted to health promotion interventions aimed at behavior change. Search results covered a broad area of behaviors, ranging from smoking cessation to mammographic screening behavior and HIV/AIDS prevention. Multiple other areas could have been addressed, as cognitions are also known to precede, for instance, mental health states like depression. Including these studies would open a whole new area in which another range of cognitive outcome measures precede the final outcome (in this case mental health). However, due to its explorative character the scope of our review was limited to health promotion interventions aimed at behavior change, as delayed behavior change and studies on cognitive outcome measures are common and well known in this area. Second, studies reporting cost and effects separately in a disaggregated way were excluded from this review as such studies did not report an economic evaluation. However, such studies may provide additional information on the availability of studies with data that are, in principle, suitable for the proposed frameworks in this review.

## Conclusions

From CEA literature in the field of health promotion two different frameworks were obtained that attempt to account for the complex process of behavior change in CEAs of behavioral interventions. Both frameworks assume that changes in cognitions, as antecedents of behavior, are predictive of future behavioral change. In the first approach, cognitive outcome measures were modeled to predict future behavior change and included in the ICER. In the second approach cognitive outcome measures were presented as effectiveness measures in CEAs. Importantly, CEAs that do consider cognitive outcome measures in their methods show that CEA may not capture the full impact of interventions if partial behavior change is not considered. This can be based on the different ICERs found in analyses that included cognitive outcome measures, when compared to standard CEA methods that do not directly recognize the effect of behavioral change.

The present review shows that the potential value of cognitive states in CEA, as a way to account for partial behavior change, is to some extent recognized, but not (yet) integrated in the field. In conclusion, CEAs should consider, and where appropriate incorporate measures of partial behavioral change when reporting effectiveness and hence cost-effectiveness.

## Competing interests

The authors declare that they have no competing interests.

## Authors' contributions

RP, LMAB en MEP developed the method for this study and analyzed the results. RP wrote the paper. All the authors provided significant comments, read and approved the final manuscript.
